# Predictors of response in PROMIS-global in a chronic low back pain specialty clinic: STarTBack and chronic overlapping pain conditions

**DOI:** 10.3233/BMR-230067

**Published:** 2024-07-02

**Authors:** Patricia Zheng, Susan Ewing, Angelina Tang, Dennis Black, Trisha Hue, Jeffrey Lotz, Thomas Peterson, Abel Torres-Espin, Conor O’Neill

**Affiliations:** a Department of Orthopaedic Surgery, University of California, San Francisco, CA, USA; b Department of Epidemiology and Biostatistics, University of California, San Francisco, CA, USA; c School of Medicine, University of California, San Francisco, CA, USA; d Bakar Computational Health Sciences Institute, University of California, San Francisco, CA, USA; e Department of Neurological Surgery, University of California, San Francisco, CA, USA; f Department of Physical Therapy, University of Alberta, Edmonton, AB, Canada; g School of Public Health Sciences, University of Waterloo, Waterloo, ON, Canada

**Keywords:** Non-surgical spine care, precision medicine, specialty clinic, low back pain

## Abstract

**BACKGROUND:**

Tools, such as the STarTBack Screening Tool (SBT), have been developed to identify risks of progressing to chronic disability in low back pain (LBP) patients in the primary care population. However, less is known about predictors of change in function after treatment in the specialty care population.

**OBJECTIVE:**

We pursued a retrospective observational cohort study involving LBP patients seen in a multidisciplinary specialty clinic to assess which features can predict change in function at follow-up.

**METHODS:**

The SBT was administered at initial visit, and a variety of patient characteristics were available in the chart including the presence of chronic overlapping pain conditions (COPCs). Patient Reported Outcomes Measurement Information System-10 (PROMIS-10) global physical health (PH) and global mental health (MH) were measured at baseline and at pragmatic time points during follow-up. Linear regression was used to estimate adjusted associations between available features and changes in PROMIS scores.

**RESULTS:**

241 patients were followed for a mean of 17.0 ± 7.5 months. Mean baseline pain was 6.7 (SD 2.1), PROMIS-10 global MH score was 44.8 (SD 9.3), and PH score was 39.4 (SD 8.6). 29.7% were low-risk on the SBT, 41.8% were medium-risk, and 28.5% were high-risk. Mean change in MH and PH scores from baseline to the follow-up questionnaire were 0.86 (SD 8.11) and 2.39 (SD 7.52), respectively. Compared to low-risk patients, high-risk patients had a mean 4.35 points greater improvement in their MH score (p= 0.004) and a mean 3.54 points greater improvement in PH score (p= 0.006). Fewer COPCs also predicted greater improvement in MH and PH.

**CONCLUSIONS:**

SBT and the presence of COPC, which can be assessed at initial presentation to a specialty clinic, can predict change in PROMIS following treatment. Effort is needed to identify other factors that can help predict change in function after treatment in the specialty care setting.

## 1. Introduction

Low back pain (LBP) afflicts 8.2% of United States adults [1]. It is the leading global cause of disability [2]. While multiple treatments are available, their mean treatment effects are typically mild, especially for chronic low back pain [3]. Even the best treatments improve pain by only two points on an eleven-point Visual Analogue Scale (VAS) scale. LBP is a heterogeneous condition influenced by multiple factors including biological, psychological, and social factors, as well as age [4], gender [5], race [6], culture [7], and co-morbidities [8, 9]. Identifying specific subgroups that respond best to targeted treatments is a key objective for LBP research [10].

The Keele STarTBack Screening Tool (SBT) [11, 12] was developed in the United Kingdom (UK) and identifies three subgroups of low back pain patients in primary care, defined by their risk of progressing to persistent disability (low risk, medium risk, high risk). The SBT is easy to administer as it has only 9 items. Four items focus on psychosocial factors: catastrophizing, fear, anxiety, and depression [11]. These psychosocial factors were chosen as they were thought to be modifiable in the primary care setting. The SBT successfully predicts long-term disability in both UK and United States (US) primary care settings [13, 14]. In the UK population specifically, stratifying treatment based on the risk subgroup reduced disability [12]. As such, SBT is being increasingly used to guide primary care pathways for back pain [15].

While the SBT has been extensively evaluated in primary care, there is less evidence supporting its use in other settings. SBT classification at initial presentation to the Emergency Department did not predict outcome pain intensity and disability at 6 weeks or at 26 weeks [16]. Field and Newell found that SBT is not as successful in differentiating outcomes in LBP patients seeking chiropractic care in the UK [17]. In the physical therapy setting, Beneciuk found that SBT was able to predict 6-month disability outcomes but not pain in a US population of chronic low back pain patients [18]. Kendell and colleagues studied SBT in a more heterogeneous population of cLBP patients in Western Australia recruited from general community, physical therapy, psychology, and pain management clinics. They found that the SBT did predict disability but was unable to discriminate changes in pain or global perceived change on a 7-point Global Rating of Change Scale [13]. The ability of SBT to predict disability has not been studied in specialty care settings.

Other groups have looked for alternative ways to screen those at high risk for chronic disability. Some have used pre-existing surveys assessing “pain”, “distress”, “social-environment”, and “medical care-environment” [19]. However, the need to use multiple surveys to measure multiple domains can hamper the ease of use of these instruments. A potential solution, which would improve risk stratification with minimal impact on patient and provider burden, is to use information readily available in the electronic health record (EHR) alongside SBT to help predict outcome. Rodeghero and colleagues demonstrated the feasibility of such a risk stratification approach for low back pain patients in physical therapy practice. They found that insurance type, duration of symptoms, and past surgery are the strongest predictors of outcome [20].

The objective of our study was to determine what features available in the EHR at the time of initial visit, including SBT, can predict change in function in response to treatment in a low back pain specialty clinic.

**Figure 1.**
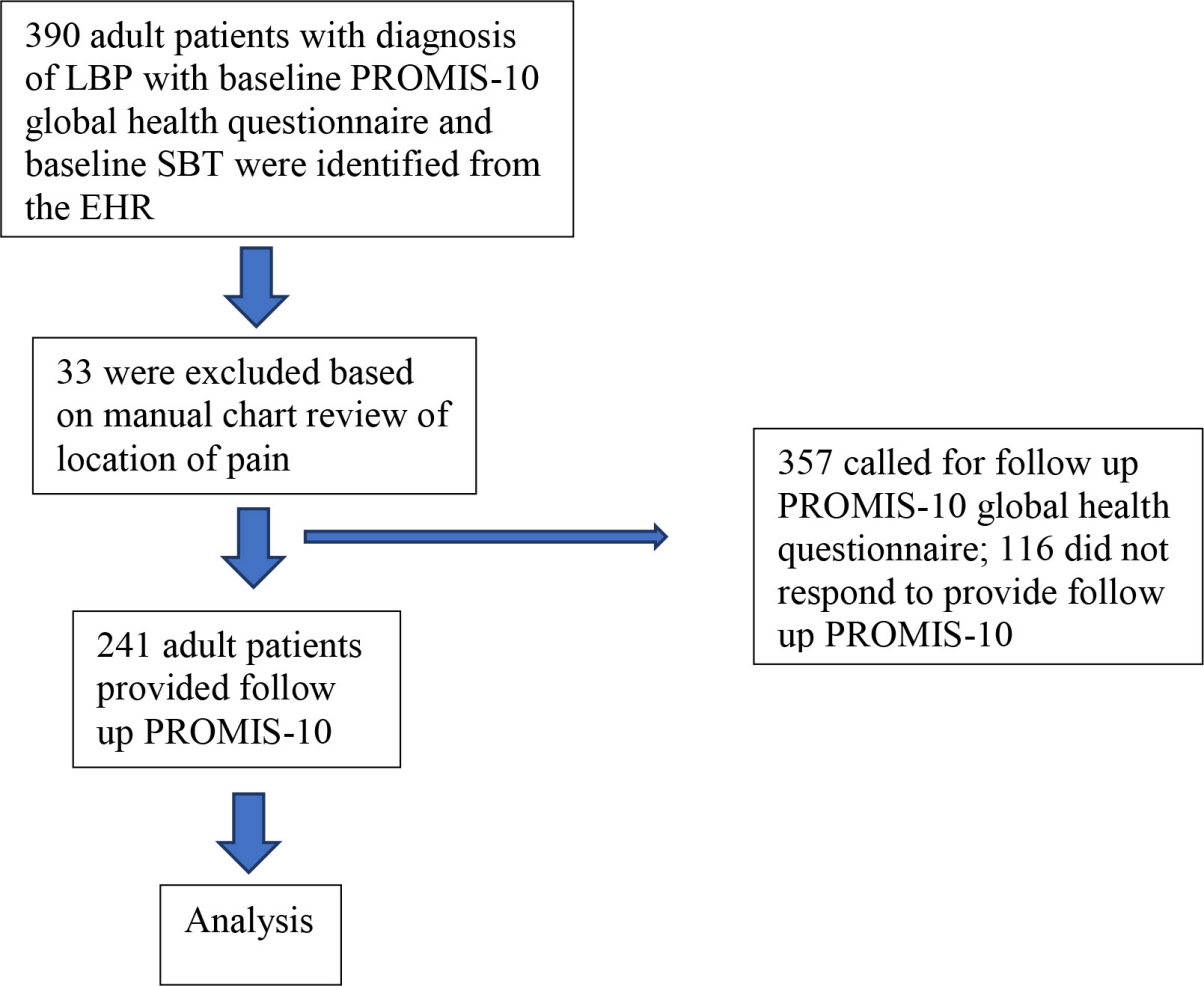
Patient flow.

## 2. Methods

### 2.1 Study design and setting

This work is a retrospective observational cohort study involving LBP patients seen in a LBP specialty clinic – the UCSF Integrated Spine Service (ISS). The Strengthening the Reporting of Observational Studies in Epidemiology (STROBE) reporting guideline for cohort studies was followed. ISS is a multidisciplinary program focused on improving the quality of care delivered to patients with spinal (cervical, thoracic, or lumbar) pain. The program is still small and growing, requires a primary care physician referral, and is restricted to patients with chronic spinal pain. Patients are scheduled for back-to-back appointments with a physical therapist and a physician (either a physiatrist or pain management specialist), who formulate a joint treatment plan. Patients receive consistent messaging about spinal pain from their providers, organized around the principles of pain neuroscience education. Self-care strategies, active rehabilitation, and non-interventional treatments are emphasized. Information from providers is reinforced by print and on-line materials. The STarT Back screening tool (SBT) is administered to all patients at baseline. There are no standardized treatment pathways, but patients who fall into the high-risk subgroup are discussed at monthly multidisciplinary case conferences so that progress is closely monitored and treatment adjusted as indicated. The study received institutional review board approval.

### 2.2 Participants

Inclusion criteria consisted of enrollment in ISS between 2018 and 2020 with documented low back pain with baseline and follow-up PROMIS-10 data. All 390 adult patients referred to ISS who completed baseline PROMIS-10 global health questionnaire and baseline SBT were identified through the EHR. LBP patients were identified through diagnosis codes [21, 22]. After manual chart review to verify location of pain (PZ, AT), 33 patients were excluded for not having LBP, leaving 357 patients. There were no other exclusion criteria for the study, though referral to ISS requires that red flag conditions (e.g., tumor, infection, neurologic deficits, fractures) have been ruled out in primary care. As part of a quality improvement project, a one-time follow-up survey was conducted via phone or email in August 2020 (PZ, AT). Included in the analysis were 241 patients who completed follow-up PROMIS-10 questions (Fig. 1).

### 2.3 Data measurement

As detailed above, the SBT was administered to all patients at baseline before admission to ISS. The NRS and PROMIS-10 global health questionnaire were administered at baseline as well. Follow-up NRS and PROMIS-10 were obtained via phone or email in August 2020 (PZ, AT). Similarly, clinical notes of individual patients were reviewed (PZ, AT) at the initial visit to characterize pain intensity (NRS), duration of pain, other pain locations, and presence of clinician diagnosed weakness. In addition, all other available features available within the electronic health record (EHR) at time of referral were collected. This included using ICD-10 codes available in the chart to assess the patient’s Charlson Comorbidity Index (CCI) [23], presence of chronic overlapping pain conditions (COPCs) [9], and use of prescription medications including anti-inflammatory, analgesic, opioids, anti-convulsants, and glucocorticoids (see full list of codes in the Appendix). Similarly, utilization of imaging, emergency department visits, nerve tests, physical therapy visits, acupuncture, hospitalization, injections, and surgeries in the 6 months prior to the baseline visit (see full list of codes in the Appendix) were tabulated.

### 2.4 Statistical methods

Baseline characteristics by SBT category were summarized using means and standard deviations (SDs) for continuous measures and counts and percentages for categorical measures. Chi-square tests of homogeneity, analyses of variance, and Kruskal-Wallis tests were used to compare baseline characteristics by SBT category.

Change in PROMIS scores were normally distributed. Linear regression models were used to determine the associations between baseline characteristics and change in PROMIS scores, with results presented as the mean difference and 95% confidence interval (CI) in change in PROMIS scores for a given change in baseline characteristic. Multivariate models included characteristics that were associated with change in PROMIS scores at p-value < 0.10 in unadjusted models. All analyses were performed with SAS software (version 9.4, SAS Institute Inc., Cary, NC, USA).

## 3. Results

**Table 1:** Baseline characteristics of ISS patients

Characteristic	All		STarTBack risk assessment^*^						
	participants (n= 241)		Low (n= 71)		Medium (n= 100)		High (n= 68)		p-value
NRS score	6.7	± 2.1	5.4	± 2.0	7.0	± 2.1	7.5	± 1.8	< 0.0001
PROMIS MH score	44.8	± 9.4	50.2	± 8.0	45.9	± 8.4	38.0	± 7.7	< 0.0001
PROMIS PH score	39.4	± 8.6	46.7	± 7.5	38.8	± 6.4	33.0	± 7.0	< 0.0001
Age (years)	59.5	± 16.4	57.9	± 17.5	61.0	± 15.7	59.3	± 16.4	0.47
Female	148	(61.7)	36	(50.7)	63	(63.0)	47	(70.2)	0.06
Hispanic	20	(8.5)	7	(10.3)	6	(6.2)	7	(10.3)	0.54
Race									0.11
Caucasian	104	(44.0)	28	(41.2)	53	(54.1)	23	(33.8)	
African American	33	(14.0)	9	(13.2)	10	(10.2)	13	(19.1)	
Asian	67	(28.4)	22	(32.4)	20	(20.4)	24	(35.3)	
Other	32	(13.6)	9	(13.2)	15	(15.3)	8	(11.8)	
Weight (lb)	169.5	± 40.0	159.6	± 32.4	174.1	± 38.4	172.4	± 47.2	0.051
Height (cm)	166.7	± 9.9	167.3	± 8.3	168.0	± 10.5	164.0	± 10.4	0.03
BMI (kg/m^2^)	27.6	± 5.8	25.9	± 4.9	27.8	± 5.4	28.9	± 6.7	0.008
Health insurance type									0.0001
Private	104	(43.2)	38	(53.5)	46	(46.0)	19	(27.9)	
Medicare	92	(38.2)	29	(40.9)	38	(38.0)	25	(36.8)	
Medi-cal	45	(18.7)	4	(5.6)	16	(16.0)	24	(35.3)	
Smoking status									0.42
Everyday	6	(2.5)	0	(0)	3	(3.0)	3	(4.4)	
Occasionally	3	(1.3)	0	(0)	1	(1.0)	2	(2.9)	
Past	88	(36.5)	25	(35.2)	39	(39.0)	24	(35.3)	
Never	144	(59.8)	46	(64.8)	57	(57.0)	39	(57.4)	
Worst pain location: lower back	184	(77.3)	45	(64.3)	80	(80.8)	58	(86.6)	0.005
Duration of pain									0.22
⩽ 1 year	83	(39.7)	32	(51.6)	32	(36.0)	18	(31.6)	
1–5 years	42	(20.1)	10	(16.1)	19	(21.4)	13	(22.8)	
> 5 years	84	(40.2)	20	(32.3)	38	(42.7)	26	(45.6)	
Count of other pain locations	1.2	± 1.1	1.0	± 1.0	1.1	± 1.1	1.6	± 1.2	0.009
Widespread pain	157	(68.9)	44	(65.7)	64	(66.0)	49	(79.0)	0.16
Pain down buttock/thigh	94	(44.3)	19	(29.2)	46	(54.8)	29	(47.5)	0.007
Pain down knee	51	(24.9)	8	(12.7)	24	(30.4)	19	(31.2)	0.02
Pain down leg (buttock/thigh or knee)	95	(44.8)	19	(29.2)	46	(54.8)	30	(49.2)	0.006
Weakness (strength < 4 out of 5)	11	(4.6)	2	(2.8)	4	(4.0)	5	(7.4)	0.41
Charlson Comorbidity Index score	1.3	± 1.3	1.1	± 1.2	1.3	± 1.5	1.3	± 1.3	0.74
Charlson Comorbidity Index category									0.64
CCI score 0	86	(35.7)	24	(33.8)	39	(39.0)	22	(32.4)	
CCI score 1–2	118	(49.0)	39	(54.9)	42	(42.0)	36	(52.9)	
CCI score 3–4	30	(12.4)	6	(8.5)	16	(16.0)	8	(11.8)	
CCI score 5+	7	(2.9)	2	(2.8)	3	(3.0)	2	(2.9)	
Count of chronic overlapping pain conditions	0.25	± 0.50	0.11	± 0.32	0.30	± 0.58	0.32	± 0.53	0.02
Overlapping pain conditions in previous 6 months	52	(21.6)	8	(11.3)	24	(24.0)	20	(29.4)	0.03
Analgesic/anti-inflammatory use in previous 6 months	109	(45.4)	26	(37.1)	39	(39.0)	42	(61.8)	0.004
Opioid use in previous 6 months	98	(40.8)	22	(31.4)	42	(42.0)	33	(48.5)	0.12
Other anesthetic/glucocorticoid/cannabinoid use in previous 6 months	53	(22.1)	11	(15.7)	20	(20.0)	21	(30.9)	0.08
Other anti-convulsant/non-benzodiazepine use in previous 6 months	65	(27.1)	12	(17.1)	30	(30.0)	22	(32.4)	0.09
Benzodiazepine use in previous 6 months	4	(1.7)	2	(2.9)	2	(2.0)	0	(0)	0.40
Anti-depressant use in previous 6 months	22	(9.2)	3	(4.3)	6	(6.0)	13	(19.1)	0.004
Imaging in previous 6 months	75	(31.1)	19	(26.8)	34	(34.0)	22	(32.4)	0.59
Outpatient visit in previous 6 months	234	(97.9)	69	(97.2)	97	(97.0)	68	(100)	0.36
Emergency department visit in previous 6 months	36	(14.9)	10	(14.1)	12	(12.0)	12	(17.7)	0.59
Nerve tests in previous 6 months	8	(3.3)	2	(2.8)	5	(5.0)	1	(1.5)	0.44
Physical therapy in previous 6 months	38	(15.8)	11	(15.5)	16	(16.0)	10	(14.7)	0.97
Inpatient hospitalization in previous 6 months	2	(0.8)	0	(0)	0	(0)	1	(1.5)	0.28
Injections in previous 6 months	8	(3.3)	3	(4.2)	3	(3.0)	2	(2.9)	0.89
Acupuncture in previous 6 months	6	(2.5)	0	(0)	3	(3.0)	3	(4.4)	0.23
Surgery in previous 6 months	1	(0.4)	0	(0)	1	(1.0)	0	(0)	0.50

Data presented as mean ± SD or n (%). ^*^Two participants did not have SBT data (hence the difference in 239 vs. 241).

Baseline characteristics among all 241 patients and by SBT category are summarized in Table 1. Overall, the mean age (± SD) was 59.5 ± 16.4 years, 61.7% were Female, 8.5% were Hispanic, with 44.0% being Caucasian, 14.0% African American, 28.4% Asian and 13.6% other race. About one-third of patients (29.7%) were low risk on the SBT, 41.8% were medium risk, and 28.5% were high risk. Average pain at time of starting ISS was 6.7 ± 2.1 per the numerical rating scale (NRS). The average baseline PROMIS mental health score was 44.8 ± 9.4, and the average baseline PROMIS physical health score was 39.4 ± 8.6. The average Charlson Comorbidity Index (CCI) was 1.3 ± 1.3, and the average number of COPCs was 0.25 ± 0.50, with 21.6% having at least one overlapping pain condition in the previous 6 months. The most common COPCs were migraine (11.2%), irritable bowel syndrome (4.1%), fibromyalgia (2.9%), tension headaches (2.5%), and endometriosis (2.1%).

Compared to low-risk and medium-risk patients, high-risk SBT patients were more likely to have increased pain and worse mental and physical health function at baseline (Table 1). High-risk SBT patients also had higher mean BMI and were more likely to be on Medi-cal and to have used analgesics and anti-depressants in the 6 months prior to baseline.

**Table 2:** Unadjusted mean difference in change in PROMIS score for given change in demographics

			Change in PROMIS mental score (n= 238) Overall mean change: +0.86		Change in PROMIS physical score (n= 240) Overall mean change: +2.39	
Characteristic	Mean ± SD or prevalence	Unit/referent^#^	Difference (95% CI)		Difference (95% CI)	
Age (years)	59.5 ± 16.4	+5 years	-0.05	(-0.36, 0.26)	-0.14	(-0.43, 0.14)
Female	61.7%	Male	0.34	(-1.79, 2.47)	-0.46	(-2.42, 1.50)
Hispanic	8.5%	Non-hispanic	0.04	(-3.67, 3.75)	0.17	(-3.31, 3.65)
Race	44.1%	Caucasian				
African American	14.0%		3.12	(-0.03, 6.28)	0.41	(-2.59, 3.41)
Asian	28.4%		-1.82	(-4.31, 0.66)	-1.89	(-4.21, 0.44)
Other	13.6%		0.69	(-2.50, 3.88)	-1.24	(-4.25, 1.76)
Weight (lb)	169.5 ± 40.0	+40.0 lb	0.56	(-0.47, 1.59)	0.55	(-0.39, 1.50)
Height (cm^2^)	166.7 ± 9.9	-9.9 cm^2^	-0.01	(-1.13, 1.10)	-0.62	(-1.64, 0.40)
BMI (kg/m^2^)	27.6 ± 5.8	+5.8 kg/m^2^	0.43	(-0.59, 1.45)	0.11	(-0.84, 1.06)
Health insurance type	43.2%	Private				
Medicare	38.2%		0.05	(-2.25, 2.35)	0.15	(-1.97, 2.28)
Medi-cal	18.7%		0.23	(-2.64, 3.11)	0.91	(-1.73, 3.54)
Smoking status	59.8%	Never				
Everyday	2.5%		8.96	(2.40, 15.53)^**^	7.67	(1.58, 13.76)^**^
Occasionally	1.2%		-2.23	(-11.41, 6.96)	3.08	(-5.45, 11.61)
Past	36.5%		0.09	(-2.04, 2.23)	0.07	(-1.91, 2.05)

^#^For categorical variables, the referent group is listed. For weight, height, and BMI, units are per 1 SD. ^* P ⩽^ 0.05; P**⩽ 0.01.

**Table 3:** Unadjusted mean difference in change in PROMIS score for given change in pain characteristics

			Change in PROMIS mental score (n= 238) Overall mean change: +0.86		Change in PROMIS physical score (n= 240) Overall mean change: +2.39	
Characteristic	Mean ± SD or prevalence	Unit/referent#	Difference (95% CI)		Difference (95% CI)	
Worst pain location: lower back	77.3%	Worst pain location not lower back	1.66	(-0.81, 4.14)	-0.91	(-3.20, 1.39)
Duration of pain	39.7%	⩽ 1 year				
1–5 years	20.1%		-0.76	(-3.82, 2.30)	-0.29	(-3.11, 2.53)
> 5 years	40.2%		0.39	(-2.13, 2.90)	0.38	(-1.92, 2.69)
Count of other pain locations	1.2 ± 1.1	+1	-0.54	(-1.47, 0.39)	-0.65	(-1.49, 0.18)
Widespread pain	68.9%	No	-0.96	(-3.23, 1.31)	-1.11	(-3.15, 0.94)
Pain down buttock/thigh	44.3%	No	1.69	(-0.54, 3.92)	2.57	(0.55, 4.60)^**^
Pain down knee	24.9%	No	2.68	(0.09, 5.26)^*^	1.99	(-0.43, 4.41)
Pain down leg (butt/thigh or knee)	44.8%	No	1.83	(-0.40, 4.05)	2.53	(0.51, 4.56)^**^
Weakness (strength < 4 out of 5)	4.6%	No	-0.36	(-5.28, 4.56)	0.37	(-4.19, 4.93)
StartBack risk assessment	29.7%	Low risk				
Medium risk	41.8%		1.35	(-1.10, 3.80)	3.62	(1.36, 5.87)^**^
High risk	28.5%		4.51	(1.83, 7.18)^**^	3.60	(1.13, 6.06)^**^
Charlson Comorbidity Index score	1.3 ± 1.3	+1 point	-0.39	(-1.15, 0.38)	-0.92	(-1.62, -0.22)^**^
Charlson Comorbidity Index category	35.7%	CCI score 0				
CCI score 1–2	49.0%		-0.90	(-3.18, 1.38)	-2.50	(-4.57, -0.44)^**^
CCI score 3–4	12.5%		-0.92	(-4.31, 2.47)	-3.92	(-7.01, -0.84)^**^
CCI score 5+	2.9%		-2.21	(-8.49, 4.07)	-3.15	(-8.87, 2.57)
Count of chronic overlapping pain conditions (COPCs)	0.25 ± 0.50	+1	-3.72	(-5.71, -1.74)^**^	-3.24	(-5.08, -1.39)^**^
Overlapping pain conditions in previous 6 months	21.6%	No	-3.87	(-6.32, -1.42)^**^	-3.43	(-5.70, -1.16)^**^

^#^For categorical variables, the referent group is listed. ^* P ⩽^ 0.05; P**⩽ 0.01.

**Table 4:** Unadjusted mean difference in change in PROMIS score for given change in self-care strategies, active rehabilitation, and treatments in last 6 months

			Change in PROMIS mental score (n= 238) Overall mean change: +0.86		Change in PROMIS physical score (n= 240) Overall mean change: +2.39	
Characteristic	Mean ± SD or prevalence	Unit/referent#	Difference (95% CI)		Difference (95% CI)	
Analgesic/anti-inflammatory use in previous 6 months	45.4%	No	0.32	(-1.76, 2.40)	-1.00	(-2.91, 0.91)
Opioid use in previous 6 months	40.8%	No	0.24	(-1.87, 2.35)	-0.48	(-2.42, 1.46)
Other anesthetic/glucocorticoid/cannabinoid use in previous 6 months	22.1%	No	1.36	(-1.12, 3.84)	0.24	(-2.07, 2.55)
Other anti-convulsant/non-benzodiazepine use in previous 6 months	27.1%	No	0.66	(-1.65, 2.98)	1.47	(-0.67, 3.62)
Benzodiazepine use in previous 6 months	1.7%	No	-1.64	(-9.68, 6.40)	-2.90	(-10.33, 4.53)
Anti-depressant use in previous 6 months	9.2%	No	-1.07	(-4.64, 2.49)	0.74	(-2.56, 4.04)
Imaging in previous 6 months	31.1%	No	0.36	(-1.86, 2.59)	0.53	(-1.52, 2.59)
Outpatient visit in previous 6 months	97.9%	No	0.78	(-6.42, 7.97)	-6.30	(-12.92, 0.32)
Emergency department visit in previous 6 months	14.9%	No	0.81	(-2.07, 3.69)	-0.82	(-3.49, 1.84)
Nerve tests in previous 6 months	3.3%	No	-3.33	(-9.43, 2.77)	-1.32	(-6.63, 3.98)
Physical therapy in previous 6 months	15.8%	No	0.75	(-2.07, 3.56)	-0.37	(-2.98, 2.24)
Inpatient hospitalization in previous 6 months	0.8%	No	4.07	(-7.23, 15.37)	1.52	(-8.96, 12.00)
Injections in previous 6 months	3.3%	No	0.65	(-5.08, 6.38)	6.89	(1.65, 12.12)^**^
Acupuncture in previous 6 months	2.5%	No	-1.96	(-8.54, 4.62)	-1.80	(-7.90, 4.29)
Surgery in previous 6 months	0.4%	No	1.65	(-14.31, 17.61)	7.94	(-6.82. 22.70)

^#^For categorical variables, the referent group is listed. ^* P ⩽^ 0.05; P**⩽ 0.01.

**Table 5:** Adjusted mean difference in change in PROMIS Mental Score for given change in baseline characteristic

Baseline characteristic	Mean ± SD	Unit/reference#	Mean difference (95% CI)			
	or prevalence		*Overall mean change: +0.86 ± 8.11*			
			Unadjusted (n= 238)		Multivariate-adjusted (n= 196)	
STarTBack risk assessment	29.7%	Low risk				
Medium risk	41.8%		1.35	(-1.10, 3.80)	1.50	(-1.22, 4.23)
High risk	28.5%		4.51	(1.83, 7.18)^**^	4.35	(1.47, 7.23)^**^
Number of chronic overlapping pain conditions	0.25 ± 0.50	+1	-3.72	(-5.71, -1.74)^**^	-4.23	(-6.35, -2.10)^**^
Pain down knee	24.9%	No	2.68	(0.09, 5.26)^*^	2.22	(-0.31, 4.76)
Race	44.1%	Caucasian				
African American	14.0%		3.12	(-0.03, 6.28)	0.19	(-3.28, 3.67)
Asian	28.4%		-1.82	(-4.31, 0.66)	-2.53	(-5.31, 0.26)
Other	13.6%		0.69	(-2.50, 3.88)	-0.05	(-3.52, 3.41)
Smoking status	59.8%	Never				
Everyday	2.5%		8.96	(2.40, 15.53)^**^	6.67	(-0.68, 14.02)
Occasionally	1.2%		-2.23	(-11.41, 6.96)	-4.51	(-15.44, 6.42)
Past	36.5%		0.09	(-2.04, 2.23)	-0.61	(-3.03, 1.81)

^#^For categorical variables, the referent group is listed. P*⩽ 0.05; P**⩽ 0.01.

During an average of 17.0 ± 7.5 months between the baseline and follow-up visits, mean change in PROMIS mental health (MH) score was +0.86 (SD = 8.11), and mean change in PROMIS physical health (PH) score was +2.39 (SD = 7.52). Unadjusted associations between change in PROMIS scores and demographics (Table 2), pain characteristics (Table 3), and care-seeking behavior (Table 4) are shown. Baseline EHR features associated with a change in MH at p-value < 0.10 in unadjusted models are shown in Table 5. Compared to patients identified as low risk on the SBT, high-risk patients had an unadjusted mean 4.51 points greater improvement in their MH score (p= 0.001). On average, each additional chronic overlapping pain condition resulted in 3.72 points less improvement in the MH score (p= 0.0003). Compared to patients who never smoked, everyday smokers had, on average, a 8.96 points greater improvement in their MH score (p= 0.008). Patients with pain down the knee had a mean 2.68 points greater improvement in their MH score compared to those without knee pain (p= 0.04). When including all measures in Table 4in the same model, two features remained significant: 1) compared to low-risk SBT patients, high-risk patients had an adjusted mean 4.35 points greater improvement in their MH score (p= 0.004); and 2) on average, each additional chronic overlapping pain condition resulted in 4.23 points less improvement in the MH score (p= 0.0001).

**Table 6:** Adjusted mean difference in change in PROMIS Physical Score for given change in baseline characteristic

Baseline characteristic	Mean ± SD	Unit/reference^#^	Mean Difference (95% CI)			
	or prevalence		*Overall mean change: +2.39 ± 7.52*			
			Unadjusted (n= 240)		Multivariate-adjusted (n= 209)	
STarTBack risk assessment	29.7%	Low risk				
Medium risk	41.8%		3.62	(1.36, 5.87)^**^	3.82	(1.50, 6.14)^**^
High risk	28.5%		3.60	(1.13, 6.06)^**^	3.54	(1.03, 6.05)^**^
Number of chronic overlapping pain conditions	0.25 ± 0.50	+1	-3.24	(-5.08, -1.39)^**^	-4.02	(-5.87, -2.17)^**^
Pain down buttock/thigh	44.3%	No	2.57	(0.55, 4.60)^*^	2.15	(0.24, 4.06)^*^
Charlson Comorbidity Index score	1.3 ± 1.3	+1	-0.92	(-1.62, -0.22)^*^	-1.06	(-1.78, -0.34)^**^
Smoking status	59.8%	Never				
Everyday	2.5%		7.67	(1.58, 13.76)^*^	6.64	(0.46, 12.82)^*^
Occasionally	1.2%		3.08	(-5.45, 11.61)	7.13	(-2.50, 16.75)
Past	36.5%		0.07	(-1.91, 2.05)	-0.51	(-2.47, 1.45)
Injections in previous 6 months	3.3%	No	6.89	(1.65, 12.12)^*^	9.31	(2.47, 16.15)^**^
Outpatient visit in previous 6 months	97.9%	No	-6.30	(-12.92, 0.32)	-5.20	(-11.32, 0.92)

^#^For categorical variables, the referent group is listed. P*⩽ 0.05; P**⩽ 0.01.

Baseline features associated with a change in PH at p-value < 0.10 in unadjusted models are shown in Table 6. When all features were included in the same model, all measures found to be significant in the unadjusted models remained significant in the multivariate models. Compared to patients identified as low risk on the SBT, medium-risk patients had an adjusted mean 3.82 points greater improvement (p= 0.002) and high-risk patients had a mean 3.54 points greater improvement (p= 0.006) in their PH score. On average, each additional chronic overlapping pain condition resulted in 4.02 points less improvement in the PH score (p< 0.0001). For each 1-point increase in the Charlson Comorbidity Index score, there was an adjusted mean 1.06 points less improvement in the PH score (p= 0.004). Patients who had injections within the 6 months prior to baseline had an adjusted mean 9.31 points greater improvement in their PH score than other patients (p= 0.008). Patients with pain down the buttock/thigh had an adjusted mean 2.15 points greater improvement in their PH score compared to those without this pain (p=A 0.03). Compared to patients who never smoked, everyday smokers had, on average, a 6.64 points greater improvement in their PH score (p= 0.04).

## 4. Discussion

Two baseline features predicted both physical and mental health disability at follow up in LBP patients in a specialty clinic setting- the SBT and COPCs. On average, compared to SBT low-risk patients, SBT medium-risk patients had a 3.82 point greater improvement in physical health (PH), and SBT high-risk patients had a 3.54 point greater improvement in physical health (PH) and 4.35 point greater improvement in their mental health (MH) score on PROMIS-global. Each additional chronic overlapping pain condition (COPCs) resulted in an average 4.02-point decrease in PH and a 4.23 point decrease in MH scores. Other EHR features associated with greater improvement in PH scores (but not MH scores) in multivariate analysis were everyday smoking status, injections in previous 6 months, lower CCI, and having pain down the buttock/thigh.

Prior studies have shown that SBT can predict disability in patients LBP in the primary care [11, 12, 14], physiotherapy [24], as well as chiropractic [25] population. However, our study is the first to look at the utility of SBT in LBP population within a specialty clinic. As compared to Suri’s study, also in the US but in the primary care setting, our patients at baseline had higher levels of pain (6.6 here on VAS as compared to 5.4 on Numerical Rating Scale) with 29% being considered high risks per SBT as compared to 21% in their group. Similarly, the physiotherapy population also has lower rates of high risk at 19% [24]. This may be expected given that patients may be more likely to be referred to a specialty clinic should they have worse pain and higher risks.

Our study focused on correlating SBT with change in PROMIS scores rather than with baseline or with follow-up scores. Clinically, there is a need to identify risk stratification tools that would predict response to a treatment such as participation in a service like ISS. Prior pragmatic randomized trials, including two performed in the United States, have also correlated SBT to change in outcome in the primary care setting. In both trials, the intensity of care was stratified based on SBT with higher acuity patients receiving more care, similar to our approach. Such stratified approach did not necessarily result in improved outcomes after treatment [26, 27]. Our study design does not allow for any conclusions about the clinical utility of SBT in cLBP specialty care, as ISS is a psychologically informed pathway that shifted during the duration of this study rather than a standardized intervention. However, patients with higher risk scores on SBT demonstrated improved outcomes after completing this treatment, perhaps because the ISS team flagged them needing more intensive care. These higher risk patients were discussed at monthly multidisciplinary case conferences so that progress was closely monitored, and treatment adjusted as indicated; this may partly explain the greater improvement seen in the high-risk patients. The overall improvement was small (0.86 for mental health and 2.39 for physical health) and did not meet the threshold for a meaningful clinical response established by Lapin and colleagues: 2.73 (SD 6.37) for mental health score and 4.76 for physical health score [28]. This finding is somewhat similar to that found by Hill and colleagues in the trial performed in the United Kingdom by Hill and colleagues where the high-risk individuals actually had improved pain score as compared to lower risk ones [12]. Perhaps the intensified care made a difference versus those with worse baseline scores had more room to improve. Further examination is warranted to see if a stratified approach as guided by SBT can predict positive response in a LBP specialty clinic.

Aside from SBT, COPCs were also predictive for changes in PH and MH. There are several plausible explanations for why COPCs also predicted both changes in physical and mental health scores. COPCs refer a variety of pain disorders that are distinct conditions, such as headache, low back pain, fibromyalgia, temporomandibular disorders, and irritable bowel syndrome, but share common comorbidities and risk factors such as female sex, increased pain sensitivity, and genetic variants [29]. While the impact of the presence of COPCs has not been well studied in musculoskeletal pain, the presence of COPCs has been associated with worse outcomes for the treatment of other pain conditions, such as chronic migraines [30]. It has to be explored whether the presence of COPCs is also associated portends worse outcomes for LBP, as our study suggests. Our findings suggest that screening for the presence of COPCs at the time of referral to specialty care may be helpful.

Other EHR features associated with greater improvement in PROMIS were everyday smoking status, injections in previous 6 months, lower CCI, and pain down the buttock/thigh. The observed association between smoking and improved outcomes is counterintuitive but may be spurious as only 2.5% of our cohort were smokers. In general, ISS providers advise smoking cessation and possibly these individuals were motivated to stop smoking or particularly benefitted from the intensive intervention. The association between injections in the past six months and the improved outcome may represent confounding by indication. In other words, subjects who were selected for treatment with injections were also those that were likely to have the best outcomes with other treatments, perhaps due to more focal pathology. Regarding comorbidities, various studies have demonstrated the comorbidities and poor health in general are associated together and that LBP patients have increased number of comorbidities. An increased comorbidity burden has been associated with worsened outcomes for cLBP [31]. The reason for this association is unknown, but it is possible that co-morbidities lead to a sedentary lifestyle and social isolation, which may lead to worse LBP outcomes [31, 32, 33].

Our study has several limitations. The study stemmed from a retrospective quality improvement effort with non-standardized follow-up intervals, with only 241 of the 357 patients (67%) who went through ISS during the study timeframe included. This may reflect selection bias as follow-up EMR features are prone to classification bias. However, to account for this, we did perform a baseline comparison for the 241 ppts in our analysis versus the 116 ppts not in our analysis (lost to follow up) and found no differences in age, pain, PROMIS scores, or the proportion in each of the STarTBack risk categories (Supplemental Table 1). Higher risk patients per SBT had worse NRS and PROMIS scores at baseline. It is possible the larger improvements at follow up in the higher-risk patients is due to regression to the mean. We also only had access to NRS and PROMIS outcomes and would benefit from including other more sensitive outcome scales. Our study design does not allow for any causal conclusions about the clinical utility of SBT in cLBP specialty care. From here, a randomized controlled trial that includes risk stratification and standardized targeted treatments would be needed to determine the clinical utility of the SBT, with or without supplemental features, in a specialty clinic setting.

## 5. Conclusion

In conclusion, we found that SBT and the presence of COPC, which can be assessed at initial presentation to a LBP specialty clinic, predicts change in PROMIS following treatment. As identifying subgroups that will respond to targeted treatments is a crucial objective for LBP research, more effort is needed to identify other factors, even if nonmodifiable, that can help risk stratify patients. Phenotyping is the first step of developing more tailored care.

## Author contributions

PZ, AT, CON collected data. PZ, CON, TH, SE, TP and ATE helped with data cleaning and analysis. All authors helped with manuscript writing and revisions.

## Data availability statement

Data is available from the corresponding author upon request.

## Ethical approval

The study received institutional review board approval from the University of California, San Francisco (#20-31733).

## Funding

The research reported in this publication was supported by the National Institute Of Arthritis And Musculoskeletal And Skin Diseases of the National Institutes of Health (Award number U19AR076737). The content is solely the responsibility of the authors and does not necessarily represent the official views of the National Institutes of Health. Additional funding was provided by the Foundation of PM&R. 

## Informed consent

Per the institutional review board approval from the University of California, San Francisco (#20-31733), no informed consent was necessary as data was collected as part of a quality initiative project.

## Supplementary data

The supplementary files are available to download from http://dx.doi.org/10.3233/BMR-230067.

## Supplementary Material

Supplementary table
